# Supplementary material for: An Internal Marking Method for Adult Spodoptera frugiperda Smith Using an Artificial Diet Containing Calco Oil Red N-1700
Source: Insects. 2024 Jul 25;15(8):561. doi: 10.3390/insects15080561 (PMC11354658; doi:10.3390/insects15080561)
Supplement: Supplementary file 1 [file insects-15-00561-s001.zip › insects-3107388-supplementary.pdf]

Supplementary Materials

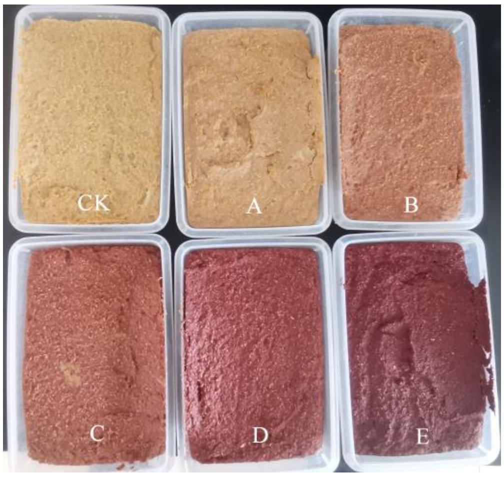

Figure S1. Artificial diets containing different concentrations of Calco Oil Red N-1700. CK: the diet without Calco Oil Red N-1700; A: the diet containing 0.01% Calco Oil Red N-1700; B: the diet containing 0.05% Calco Oil Red N-1700; C: the diet containing 0.1% Calco Oil Red N-1700; D: the diet containing 0.2% Calco Oil Red N-1700; E: the diet containing 0.5% Calco Oil Red N-1700.
